# Synergy Maps: exploring compound combinations using network-based visualization

**DOI:** 10.1186/s13321-015-0090-6

**Published:** 2015-08-01

**Authors:** Richard Lewis, Rajarshi Guha, Tamás Korcsmaros, Andreas Bender

**Affiliations:** Department of Chemistry, Centre for Molecular Informatics, University of Cambridge, Lensfield Road, Cambridge, CB2 1EW UK; National Center for Advancing Translational Sciences, 9800 Medical Center Drive, Rockville, MD 20850 USA; TGAC, The Genome Analysis Centre, Norwich Research Park, Norwich, UK; Gut Health and Food Safety Programme, Institute of Food Research, Norwich Research Park, Norwich, UK

**Keywords:** Compound combinations, Mixtures, Synergy, Visualization, Network, Dimensionality reduction

## Abstract

**Background:**

The phenomenon of super-additivity of biological response to compounds applied jointly, termed *synergy*, has the potential to provide many therapeutic benefits. Therefore, high throughput screening of compound combinations has recently received a great deal of attention. Large compound libraries and the feasibility of all-pairs screening can easily generate large, information-rich datasets. Previously, these datasets have been visualized using either a heat-map or a network approach—however these visualizations only partially represent the information encoded in the dataset.

**Results:**

A new visualization technique for pairwise combination screening data, termed “Synergy Maps”, is presented. In a Synergy Map, information about the synergistic interactions of compounds is integrated with information about their properties (chemical structure, physicochemical properties, bioactivity profiles) to produce a single visualization. As a result the relationships between compound and combination properties may be investigated simultaneously, and thus may afford insight into the synergy observed in the screen. An interactive web app implementation, available at http://richlewis42.github.io/synergy-maps, has been developed for public use, which may find use in navigating and filtering larger scale combination datasets. This tool is applied to a recent all-pairs dataset of anti-malarials, tested against *Plasmodium falciparum*, and a preliminary analysis is given as an example, illustrating the disproportionate synergism of histone deacetylase inhibitors previously described in literature, as well as suggesting new hypotheses for future investigation.

**Conclusions:**

Synergy Maps improve the state of the art in compound combination visualization, by simultaneously representing individual compound properties and their interactions. The web-based tool allows straightforward exploration of combination data, and easier identification of correlations between compound properties and interactions.

## Background

Compound combinations have recently received much interest, as they afford a number of advantages as therapeutics compared to single agent treatments across a wide range of disease areas [1–4]. The phenomenon of super-additivity of the therapeutic effect of a combination, known as *synergy*, has the potential for improved pharmaceutical treatment options in terms of increased efficacy [5] and therapeutically relevant selectivity [6], whilst reducing the risk of toxicity [7] and side-effects [8]. Two recent reviews are available on the topic [9, 10]. However, how to determine which compound combinations exhibit a desired form of synergy in a particular case is by no means clear, and the effect of multiple bioactive compounds in parallel is overall rather poorly understood.

Synergy in a combination is due to not purely additive interaction between the biological functions of the component compounds. Progress has been made in attempts to model synergy, usually by attempting to discover these interactions. For example, models incorporating flux balance analysis (FBA) have been used to correctly predict synergistic interactions in *Saccharomyces cerevisiae* [11]. Enrichment analysis of molecular and pharmacological properties predicted several combinations to be synergistic, 69% of which were subsequently verified in the literature [12]. Clinical side effect annotations have been used to predict effective combinations [13], and information from multiple domains have been integrated into a Probability Ensemble Approach to predict both efficacy and adverse effects of combinations with high predictive power [14]. Various network approaches (such as the Stochastic Block Model [15] and the Prism algorithm [15, 16]) have been used to infer novel interactions from large incomplete drug interaction databases such as DrugBank [17, 18]. Biological network topologies of drug targets that lead to synergy have been identified through network modelling [19], and mechanisms of action of many known non-additive drug combinations have been deduced [20]. However, these models usually require heavily annotated data (such as with ATC codes, protein targets or side effect data)—a complete understanding of the origins and repercussions of synergy has not yet in general been achieved, and thus significant further work is needed, both experimental and in silico.

To this end, an experimental strategy for measuring synergy has been assaying all pairwise combinations for a relatively small compound library. A recently published example of this type of dataset is the DREAM Drug Sensitivity Challenge (subchallenge 2) [21], in which all combinations of 14 compounds were tested on the LY3 lymphoma cell line. The degree of synergy for each combination was indicated by the difference in growth inhibition observed by experiment from that predicted under the Bliss Independence model [22]. Other all-pairs combinatorial datasets include a 90 compound set (consisting of drugs and probes) assayed against the HCT116 colon cancer cell line [11], a set of 11 anticancer drugs tested also tested against HCT116 [23], a set 31 antifungal compounds assayed against *S. cerevisiae* [24, 25], and an assay of 22 antibiotics against *Escherichia coli* [16]. Each of these datasets measure dose response surfaces [5], and derive synergy metrics from those surfaces (see original papers for examples). Whilst this is currently a reasonable selection in terms of dataset size, compound variety and assay type, there is potential for many more experiments—an exciting prospect is an upcoming National Cancer Institute Combination Screen of approximately 100 anti cancer drugs tested pairwise against the 59 NCI-60 cell lines [26].

### Visualizing large numbers of combinations

**Fig. 1:**
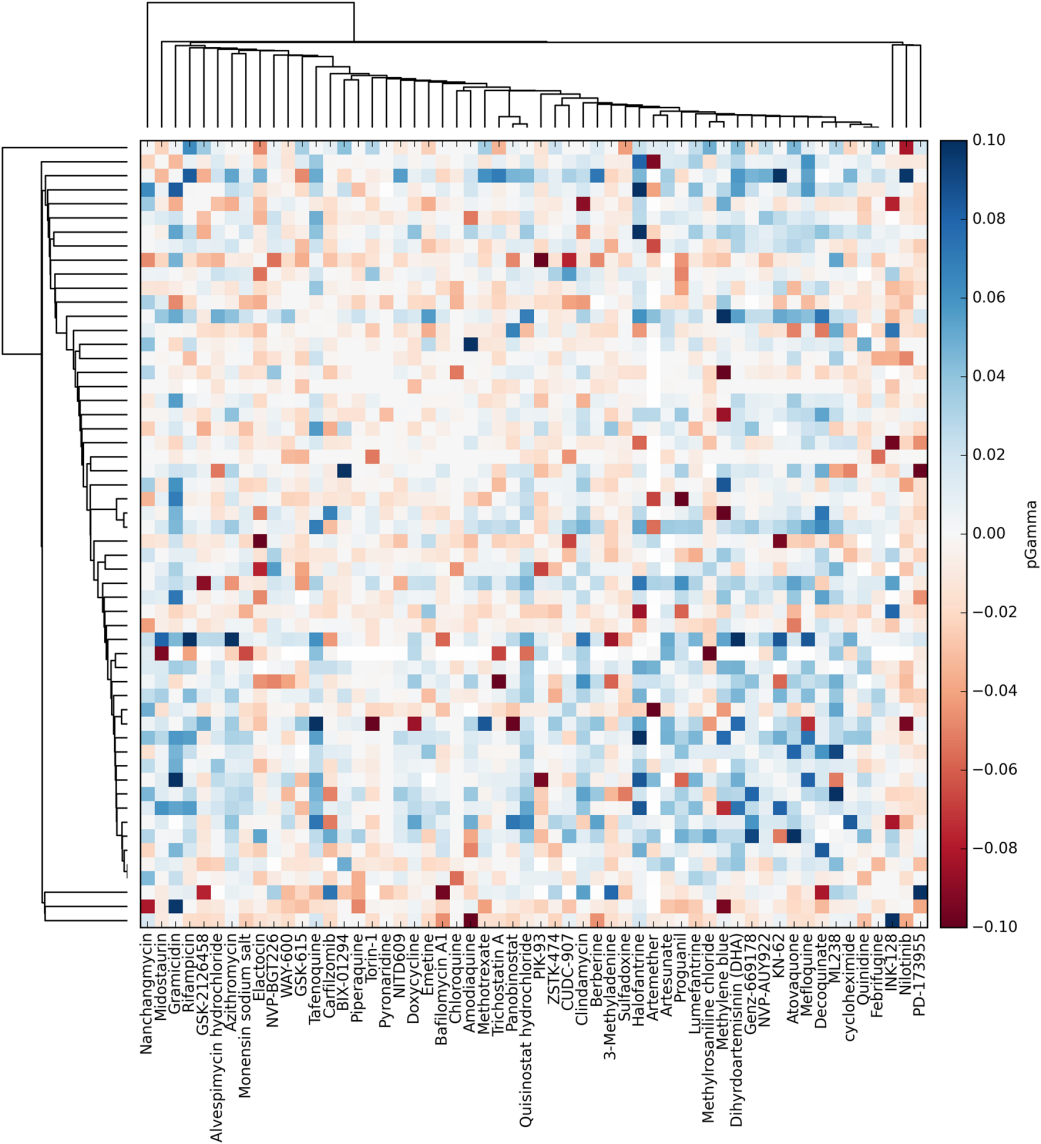
Heatmap representation of the NCATS malaria dataset. The heatmap, created using the Python visualization package matplotlib [47], is constructed as a matrix; rows and columns map to individual columns of the dataset, and the intersecting elements to their combination. The heatmap used the pGamma metric described in Table 3. Compounds were clustered according to their predicted targets, using predictions from an inhouse target prediction tool, such that compounds with a similar bioactivity profile, such as Artesunate and Artemether, cluster together.

The influx of this kind of combination data provides a new opportunity to analysts. Conventionally, a first step in a data focused study is an exploratory data analysis, principally focusing on informative visualization of any data collected with the goal of identifying major trends [27]. This can be challenging, due to structure of combination data, and the geometric scaling of possible combinations with respect to compound library size [28]. Two major approaches have been utilized to visualize combination data in the literature: *heatmaps* and *networks*. Heatmaps (see Fig. 1) are featured extensively in the literature [11, 15, 16, 21, 23, 25, 29]. Compounds of the dataset are represented as rows and columns, with their corresponding combinations positioned at the intersecting elements. A color map [11, 15, 16, 21] or gradient may be used to indicate direction and/or degree of non-additivity for each combination. The compounds may be ordered according to a particular physicochemical property, grouped by targeted protein [11] or pathway [29], hierarchically clustered according to synergy profile [25] or just alphabetically [21].

Heatmaps are useful as an uncluttered static presentation of data. It is possible to identify disproportionately synergistic compounds and also compounds that behave similarly if clustering such as in Cokol et al. [25] and Fig. 1 is applied. Additionally, relevant dose–response matrices may be superimposed [11, 23, 25] to reveal different shapes of response surfaces, which may encode information of underlying biological network topology [11, 30, 31]. A drawback is that little information about the actual compounds are encoded—they may be ordered according to a physicochemical property, but this is limits further possible insight into the dataset. Furthermore, for a large dataset (for example over a hundred compounds), such as those produced using high-throughput techniques [26], the heatmap quickly becomes cluttered and individual compounds become difficult to identify.

**Fig. 2:**
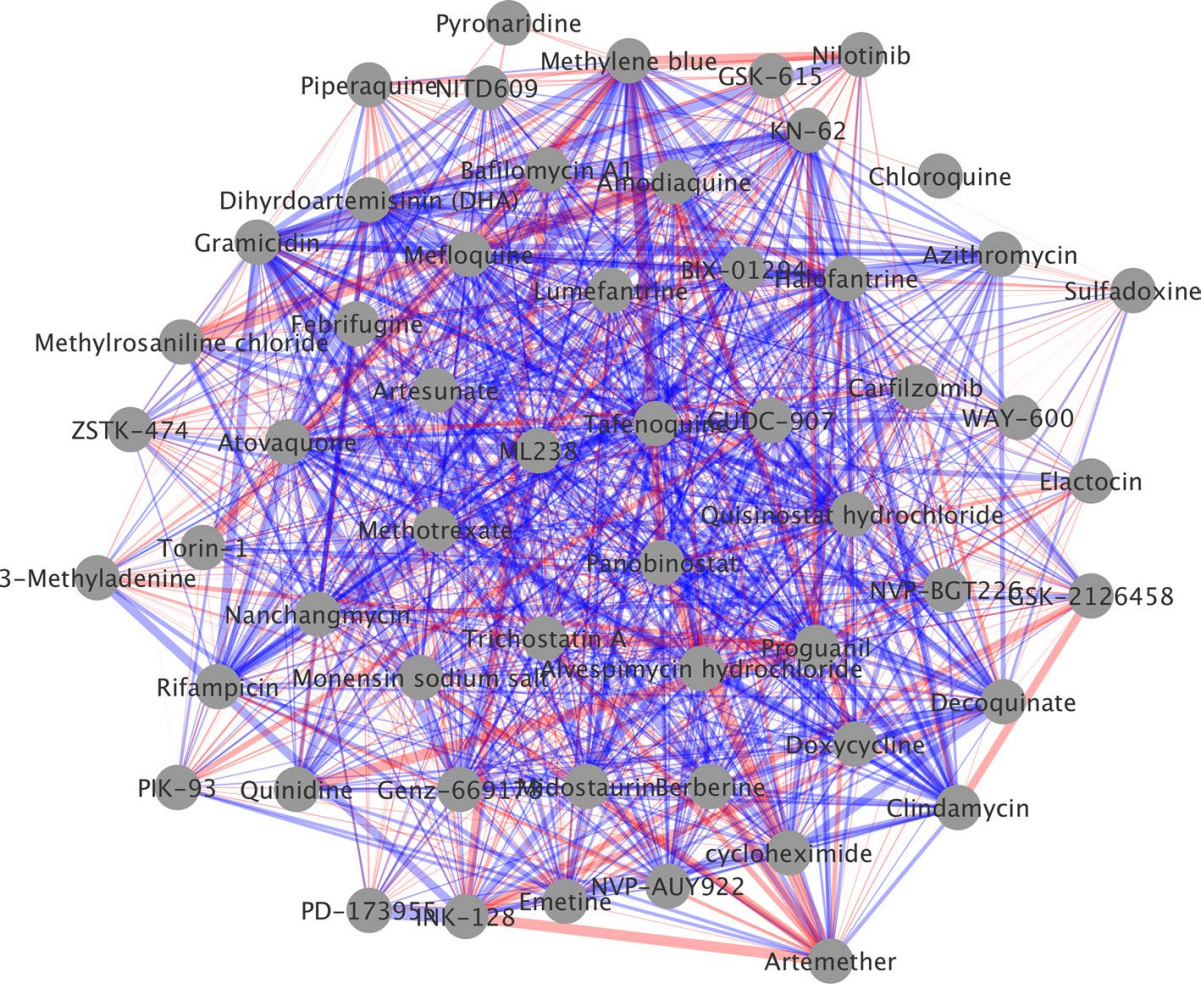
Network Representation of the NCATS malaria dataset. This network visualization was created using Cytoscape [48].* Nodes* represent compounds, whilst edges represent combinations, with thickness indicating degree of non-additivity, and red and blue indicating antagonism and synergy respectively. The layout was generated using Cytoscape’s “organic” layout routine.

Network representation (see Fig. 2) for all pairs combination data is also popular [3, 15, 16, 24, 25, 32]—nodes correspond to compounds, and edges to combinations, connecting their components. Edges may be coloured according to sign, and weighted according to degree of synergy. A graph layout algorithm, such as circular [33] or force-directed [34] is usually employed to position nodes. This type of representation has a tendency to become overcrowded, and threshold values may be required to limit the number of edges. Despite this, networks have the potential to scale better with dataset size than heatmaps as compounds are positioned in two dimensions rather than along a single one. A notable shortcoming (shared with heatmaps) is that the nature of the compounds in the dataset is not simultaneously well represented: it is only possible to show a few properties, through node color, size or superimposing numbers. An example of this may be found in a recent publication [24] where the cLogP of compounds were superimposed over the relevant node, and ordered in a circle to illustrate the increased potential of lipohilic compounds to participate in synergy. Whilst this may offer insight for the specific publication, it seems unlikely that a single property will satisfactorily explain synergistic behavior for all datasets.

Hence, an improvement in chemical property representation for the visualization of compound combination screens is still very much desirable, which is the objective of the current work.

### Chemical property visualization

Compounds have traditionally been represented under a descriptor space using a dimensionality reduction algorithm as a scatter plot; a common example is Principle Component Analysis (PCA) [35] applied to physicochemical descriptors. A state-of-the-art equivalent might be the use of Student’s t-distributed Stochastic Neighbour Embedding (t-SNE) [36] on proprietary descriptors [37]. In this way, compounds may be easily compared according to their properties or features; adjacent compounds tend to share properties and behaviour in the descriptor space in question.

In this communication, we introduce a novel type of visualization for combination datasets, named “Synergy Maps”. Synergy Maps combine network and descriptor space representation to yield an information dense presentation of a combination dataset. Specifically, the approach positions the nodes of a drug–drug interaction graph in two-dimensional space using the techniques referred to in the previous section; in this way, synergistic interactions can be straightforwardly related to trends in compound properties, and thus hypotheses for the origins of the synergy might be more quickly proposed. We also introduce an interactive implementation, which enables the generation of synergy maps for novel combination datasets, and allows for exploration of synergy under different spaces, metrics and datasets. Source code is provided as a GitHub repository.

As an example, we produce synergy maps for a combination dataset of 56 antimalarials tested against *P. falciparum*, and detail a quick analysis of the resultant maps.

## Implementation

**Fig. 3:**
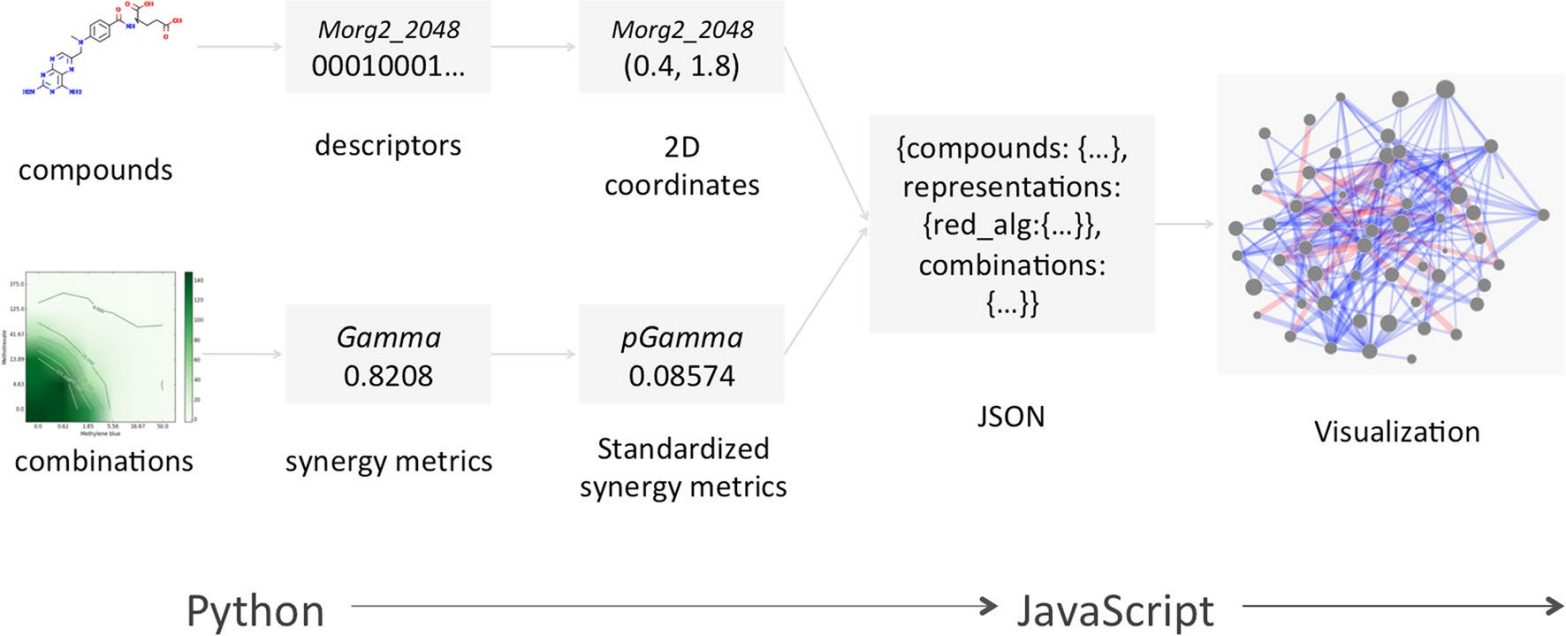
Synergy Maps work flow. The work flow employed by the Synergy Maps application. The raw compound and combination data is transformed in steps to yield processed data in JSON, which is then used by the JavaScript App to create the visualization. Specifically, the chosen descriptors (Table 1) are generated from the supplied chemical graphs, and then reduced to two dimensions by the selected dimensionality reduction techniques (Table 2). The combination data is assigned synergy values. The processed data is packaged into a JSON file.

The application was constructed according to the client–server paradigm: data processing, including the descriptor calculation, and subsequent dimensionality reduction, is performed in *Python* (the server process), then transferred via *JavaScript Object Notation* (JSON) to the client visualization, implemented in *JavaScript* (see Fig. 3 for details). The program can be run on any computer with *Python 2.7* and an HTML5 capable browser (tested on the latest *Internet Explorer*, *Safari* and *Google Chrome*).

### Data processing

An input dataset should consist of compound data in the form of a Structure-Data File (SDF), and data associated with their combinations (including calculated synergy metrics) in the form of a comma separated values (CSV) file (examples provided with the repository). A script is then written (or a default one used), specifying the descriptors, dimensionality reduction techniques and synergy metrics to employ in generating the processed file (example scripts provided with the repository).

A previously collected all pairs combination dataset of 56 compounds tested against *P. falciparum* [38] was selected as an example dataset to concretely illustrate the technique. Each combination was tested in a 6 × 6 dose–response matrix, varying the concentration of each compound on each axis. The change in growth inhibition was measured at each dose combination, yielding a response surface. From this, 9 different synergy metrics [39] were evaluated for all 1,540 combinations. These were then preprocessed into the appropriate input format.

Compounds were initially standardized using Chemaxon Standardizer [40], to ensure a consistent representation of compounds. Descriptors for each compound were calculated for physicochemical, structural and biological spaces, each of which may be of relevance to synergy (Table 1). Firstly, all available physicochemical descriptors were calculated using *PaDEL* [41]. Secondly, Morgan fingerprints of radius 2, and folded to 2,048 bits were generated as structural descriptors using *RDKit* [42]. Finally, 1080 Naive Bayes binary models, trained using ChEMBL [43] bioactivities, were used to predict likely (human) protein targets for each structure (notably, the organism of interest is not human for the example, but these descriptors act as reasonable generic biological descriptors [44]).

**Table 1:** Descriptors calculated for the compounds, which were used for later visualization in Synergy Maps

Represented space	Descriptor type	Implementation
Physicochemical	771 physicochemical descriptors	PaDEL [41]
Structural	2,048 bit folded Morgan fingerprints [49] of radius 2	RDKit [42]
Biological	1,080 bayes affinity fingerprint [44]	In house Naive Bayesian models

Three diverse spaces were selected for representation of compounds, to give maximum insight into the properties of synergy in these different spaces. Physicochemical space are likely to differentiate compounds according to their ADMET properties; structural space will differentiate compounds according to the structure of their chemical graph; finally biological space attempts to differentiate compounds according to their relative affinity for protein targets. Hence, with this selection of descriptors our software is able to highlight structure in multiple facets of a dataset.

The dimensionality of each space was then reduced to two dimensions using three different, yet complementary techniques (Table 2). Principal Component Analysis (PCA) and MultiDimensional Scaling (MDS) were run using default parameters in *scikit*-*learn* [45], and student’s t-distributed Stochastic Neighbor Embedding (t-SNE), was employed using a perplexity of 40. This yielded nine sets of coordinates per compound.

**Table 2:** Dimensionality reduction techniques implemented in Synergy Maps

Technique	Implementation
Principal Components Analysis (PCA) [35]	Scikit-learn [45]
Multidimensional Scaling (MDS)	Scikit-learn [45]
Student’s t-distributed Stochastic Neighbour Embedding (t-SNE)	According to original publication [36]

Three differing dimensionality reduction techniques were employed; these methods provide a means to interpret the approximate structure of data in extremely high dimensional space (such as physicochemical space) on a two dimensional page. PCA locates a lower dimensional hyperplane of highest variance in a hyperspace, and projects the data onto the hyperplane. MDS attempts to preserve distances in high dimensional space with those lower dimensional space. Student’s t-distributed Stochastic Neighbour Embedding also employs distance based scaling, yet imposes statistical distributions on these; it has been asserted [36] that it outperforms other methods for locating structure in high dimensional data, whilst avoiding overcrowding the centre of the low dimensional space with data points.

Due to the relatively small chemical space spanned by the 56 compounds, an additional 175 diverse compounds from MIPE [39] were temporarily added to the dataset, to diversify the space covered, and so allow for a better and more consistent dimensionality reduction step. This may not be necessary for a larger and more diverse compound set, but in practice made the resultant plots more reproducible and transferable (this was especially the case for t-SNE, which has a non-convex objective function, and thus converges to different solutions each time it is run. It also allowed for a higher perplexity (roughly the expected density of neighbors) to be set, which prevents artificially large gaps opening in the dataset).

**Fig. 4:**
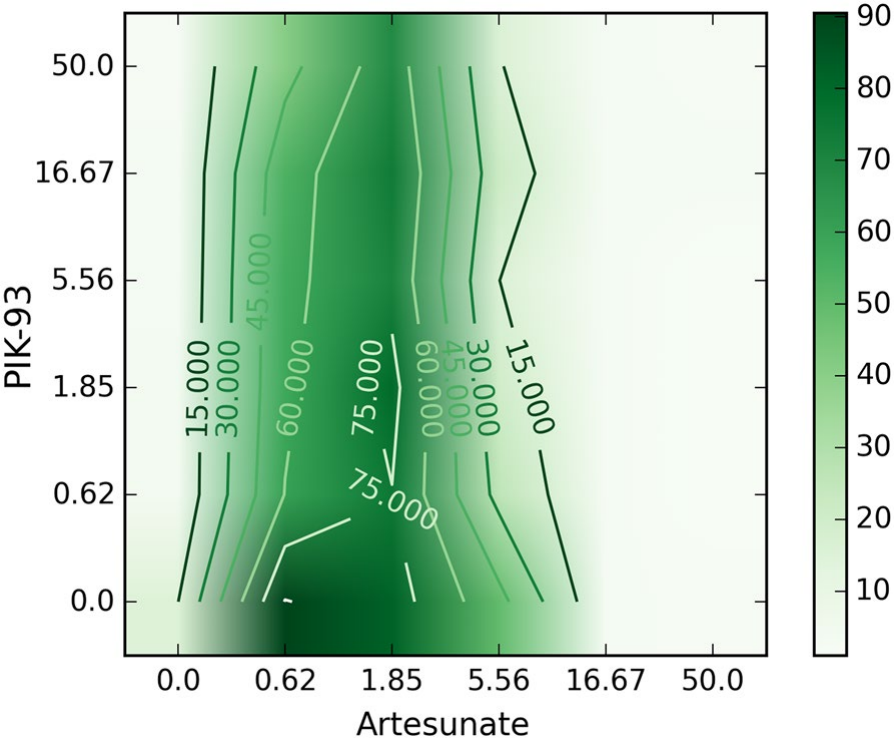
Improbable combination surface. The surface yields a suspiciously strongly antagonistic (−0.7) value of pGamma. The surface implies that the growth of *P. falciparum* is rescued by a low concentration of Artemeter, a known antimalarial. In fact, it seems much more likely that the zero concentration row has simply been contaminated, causing an incorrect value of pGamma.

The combinations were filtered for quality: firstly through the Quality Control score (removing those with a score of above 4) of the data producer [38], then by removing extreme values (top and bottom 2.5% of values sorted by Gamma) on a case by case basis, by checking whether their surfaces appeared unlikely to be genuine (for an example, see Fig. 4). The synergy metrics provided were then standardized, such that an increase in synergy was represented by an increase in magnitude, and a negative sign used for antagonism for those metrics for which it was defined (Table 3). The processed data was then outputted as a JSON formatted file.

**Table 3:** Synergy metrics calculated for the NCATS malaria dataset

Synergy metric	Derivation
pGamma	Negative based-10 logarithm of Gamma, from Cokol et al. [25]
Median Excess	The median of the sum of the differences between the combination responses and the single agent responses
Num Excess	The number of combinations in a block that show a better combination responses than both the corresponding single agents
−ExcessHSA	Negative of ExcessHSA, from Lehár et al. [11]
−ExcessCRX	Negative of ExcessCRX, from Lehár et al. [11]
LS3 × 3	The minimum value of the sum of the deviations from the HSA model are evaluated on all 3 × 3 submatrices of the response matrix (excluding the single agent row and column)
pBeta	Negative based-10 logarithm of Beta, from Cokol et al. [25]

The synergy metrics for the dataset. These were calculated for a previous study, however a couple of alterations were required to get a consistent behaviour, specifically positive and negative values relating to synergy and antagonism respectively: pBeta and pGamma were derived from Beta and Gamma [25] by taking the negative based-10 logarithm. The original metrics specify synergy below 1, and antagonism above, so this transformation handily yields the desired mapping of antagonism to negative values. −ExcessHSA and −ExcessCRX are derived from ExcessHSA and ExcessCRX [11] by taking the negative in order to attribute positive values to synergistic interactions.

### The output

The visualization stage uses the *Data Driven Documents* (D3) JavaScript library to produce a *Scalable Vector Graphics* (SVG) image of the network, positioning nodes according to the coordinates precalculated in the previous step. By default, blue and red edges represent synergistic and antagonistic combinations respectively, and edge width represents the extent of the interaction. Node area is used to represent the activity of the compound individually. Synergy cut-off values may be set using a slider to declutter the visualization of the many essentially additive combinations.

**Fig. 5:**
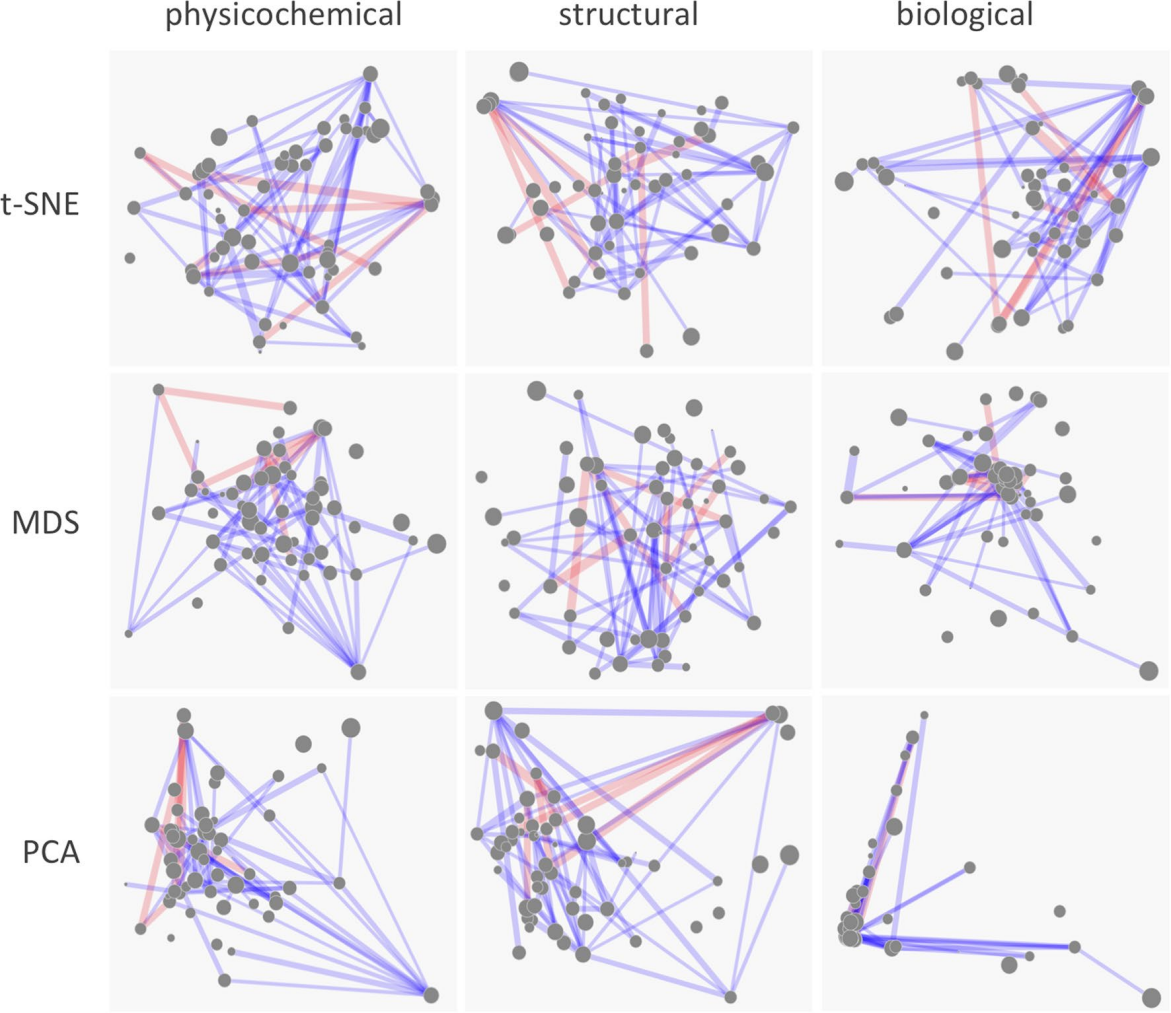
Synergy Maps. Sample static networks. Nodes represent compounds, with radius indicating relative pIC50. Edges represent combinations, with *thickness* indicating degree of non-additivity, and *red* and *blue* indicating antagonism and synergy respectively. It appears that whilst PCA is a passable dimensionality reduction algorithm for physicochemical and structural space (despite concentrating points in the centre), it does not differentiate the compounds well in biological space. MDS does a little better, yet ultimately still concentrates points towards the centre, preventing compounds from being easily being differentiated. In the authors’ opinion, t-SNE performs well in all spaces; clear clusters can be seen, identifying groups of compounds similar in that space, yet points are still spread across space helpfully so as not to clutter the visualization.

**Fig. 6:**
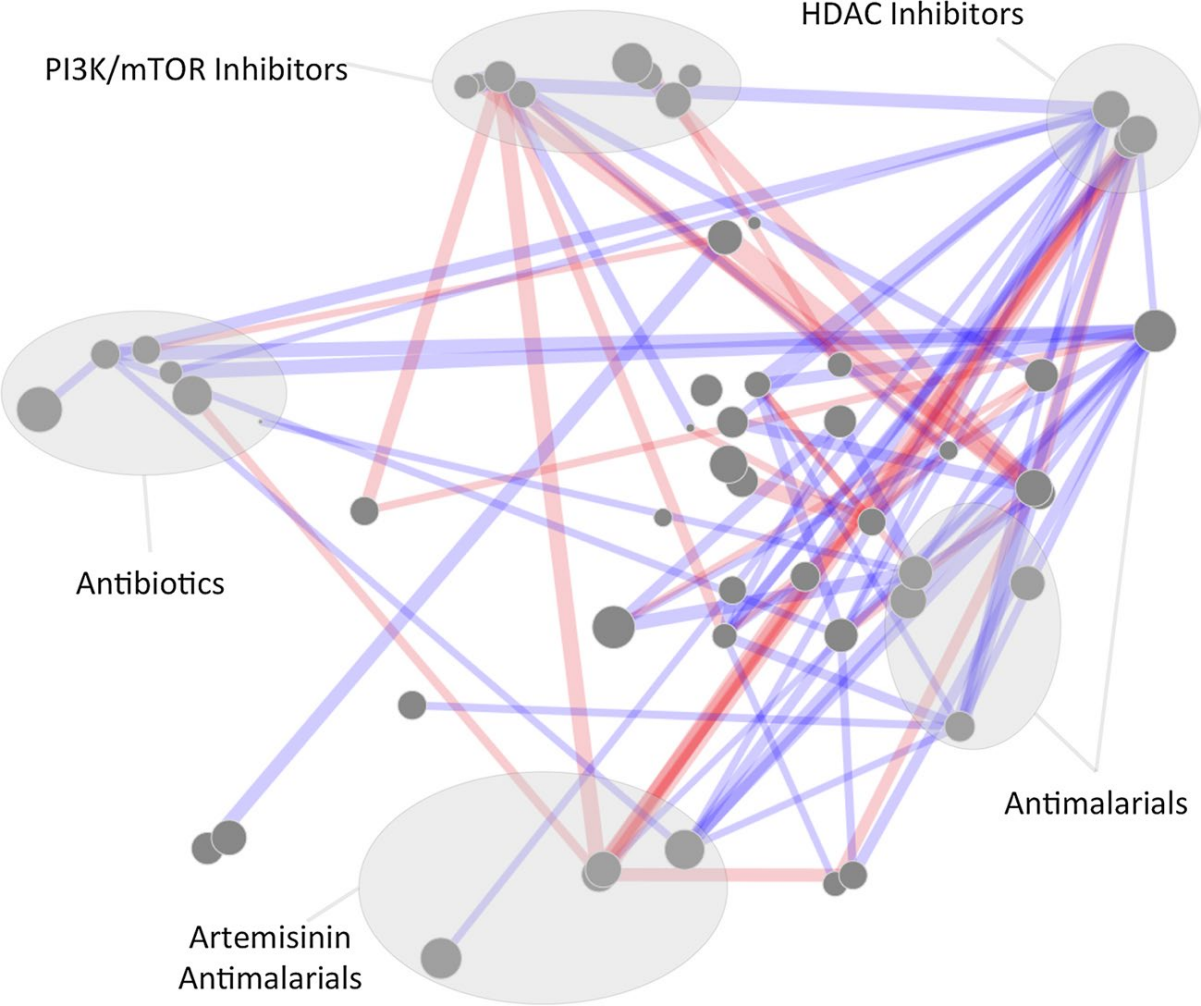
Synergy Map, represented under t-SNE reduced biological space. Biological space, with various clusters annotated according to hypothesized mode of action or drug function. It appears that the HDAC inhibitor cluster (Including Quisinostat, Trichostatin A and Panobinostat) tends to be disproportionately synergistic compared to other clusters, whilst the PI3K/mTOR inhibitors exhibit disproportional antagonism.

The resultant networks generated for the example are shown in Fig. 5, and an annotated version of t-SNE applied to the Bayes Affinity fingerprints with pGamma (negative log of the Gamma metric from Cokol et al. [25]) synergy values is shown in Fig. 6. This representation may allow for the most interesting observations to be made: compounds that are predicted to modulate similar protein targets, and thus potentially share similar modes of action, are clustered together; if similar interactions are observed consistently between clusters, the underlying modes of action of each cluster might be hypothesized to interact as the cause of the synergy.

**Fig. 7:**
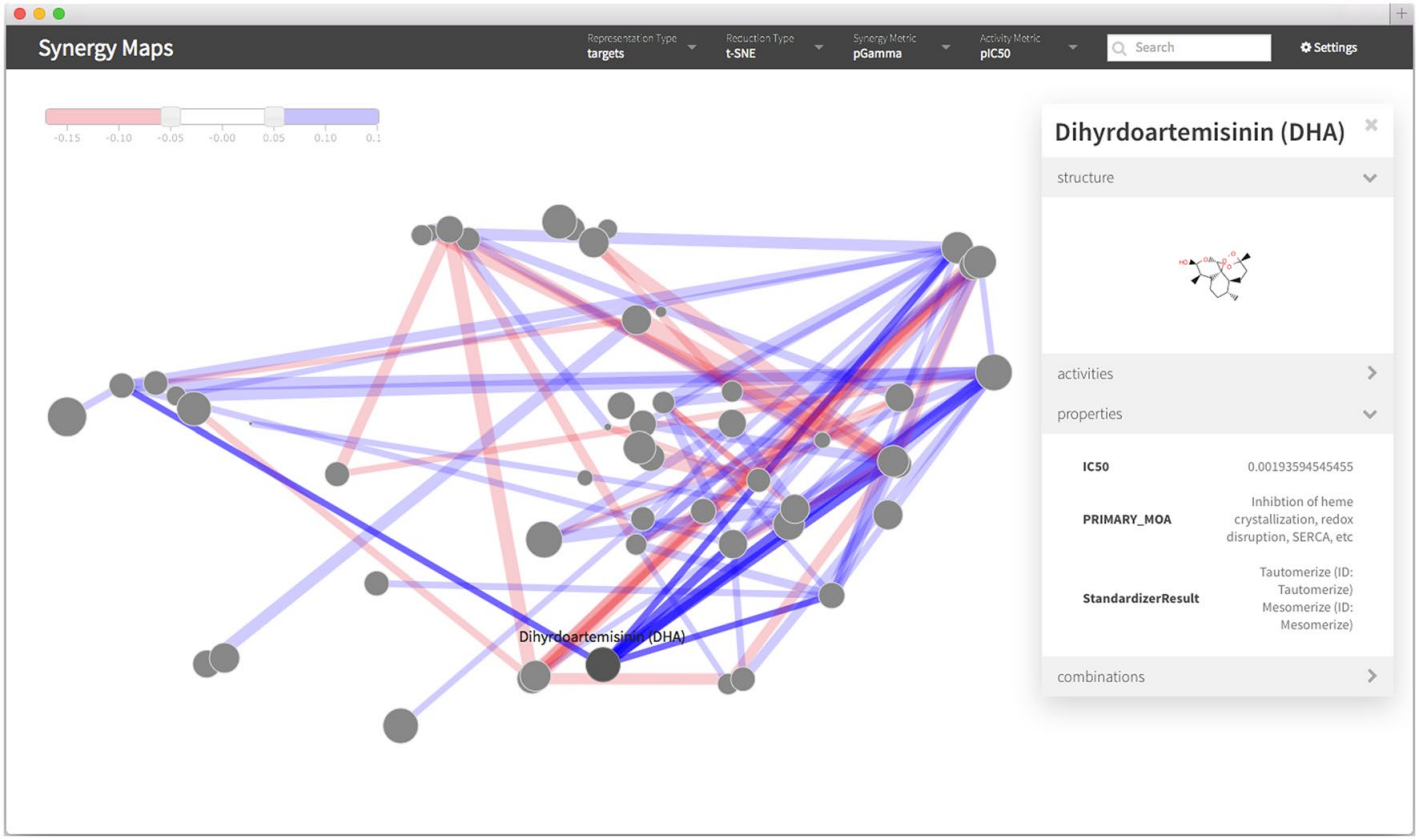
Screenshot of the interactive web visualization. A screen shot of the interactive web visualization. The representation, reduction type, synergy metric and activity metric may be set by drop down menus in the *top bar*. Compounds may be searched for using the *search box*. A slider, shown in the *top left*, may be used to select threshold levels above and below which combinations should be shown. Individual compounds and combinations may be selected to bring up a tooltip, as shown for Dihydroartemisinin in this example. The tooltip will display any extra property information supplied, such as the primary mode of action in this example. Additional metadata specific to whether a compound or a combination has been selected is also given, such as the activities for a compound, and the synergies for a combination.

The static networks provide an insight into the relationship between compound properties and synergy, but the use of *JavaScript* enabled interactivity affords more involved exploration of the data. The author’s implementation may be accessed http://atrichlewis42.github.io/synergy-maps, and source code at http://github.com/richlewis42/synergy-maps. A screen shot of the software is shown in Fig. 7. Controls allow for the synergy metric, descriptor space or dimensionality reduction technique to be changed dynamically. This feature may be used to gain a feel of the relatedness of the different synergy metrics selected, or the different spaces and reduction algorithms. A filter controlling the minimum synergy and antagonism required for display is provided to avoid overcrowding of the visualization. Tooltips provide additional information for compounds and combinations, originally supplied as extra fields in the original files; for the dataset used in this paper, an example is “hypothesized mode of action” for compounds.

## Results and discussion

Whilst the purpose of this paper is simply to introduce a novel visualization technique rather than analyze the resulting networks, it is possible to illustrate a few observations that may be made; these could be investigated further in subsequent assays. Firstly, we can see that compounds annotated as histone deacetylase (HDAC) inhibitors, which are clustered in the north-east of the Fig. 6, appear to be the most likely compounds in the dataset to be synergistic, and specifically with the compounds in the center (these are annotated with diverse modes of action, but often were kinase or phosphatase inhibitors). This property has been reported in the literature, where the HDAC inhibitor trichostatin A was found to interact synergistically with geldanamycin, an Hsp90 inhibitor [46], in *P. falciparum*. Interestingly, NVP-AUY922, an Hsp90 inhibitor included in the dataset, clustered to the centre; this is likely where geldanamycin would also be placed due to their similar annotated modes of action. This result would be in agreement with the observed trend and suggest that the method might yield some predictive power for unknown combinations. In contrast to this, PI3K inhibitors are shown to exhibit in general disproportionately more antagonism with the other compounds in the dataset. Whilst these observations are by no means reliable by themselves, they may form a basis for further study, and provide an example in how this type of visualization may prove a useful first step in the analysis of pairwise combination data.

## Comparisons

**Fig. 8:**
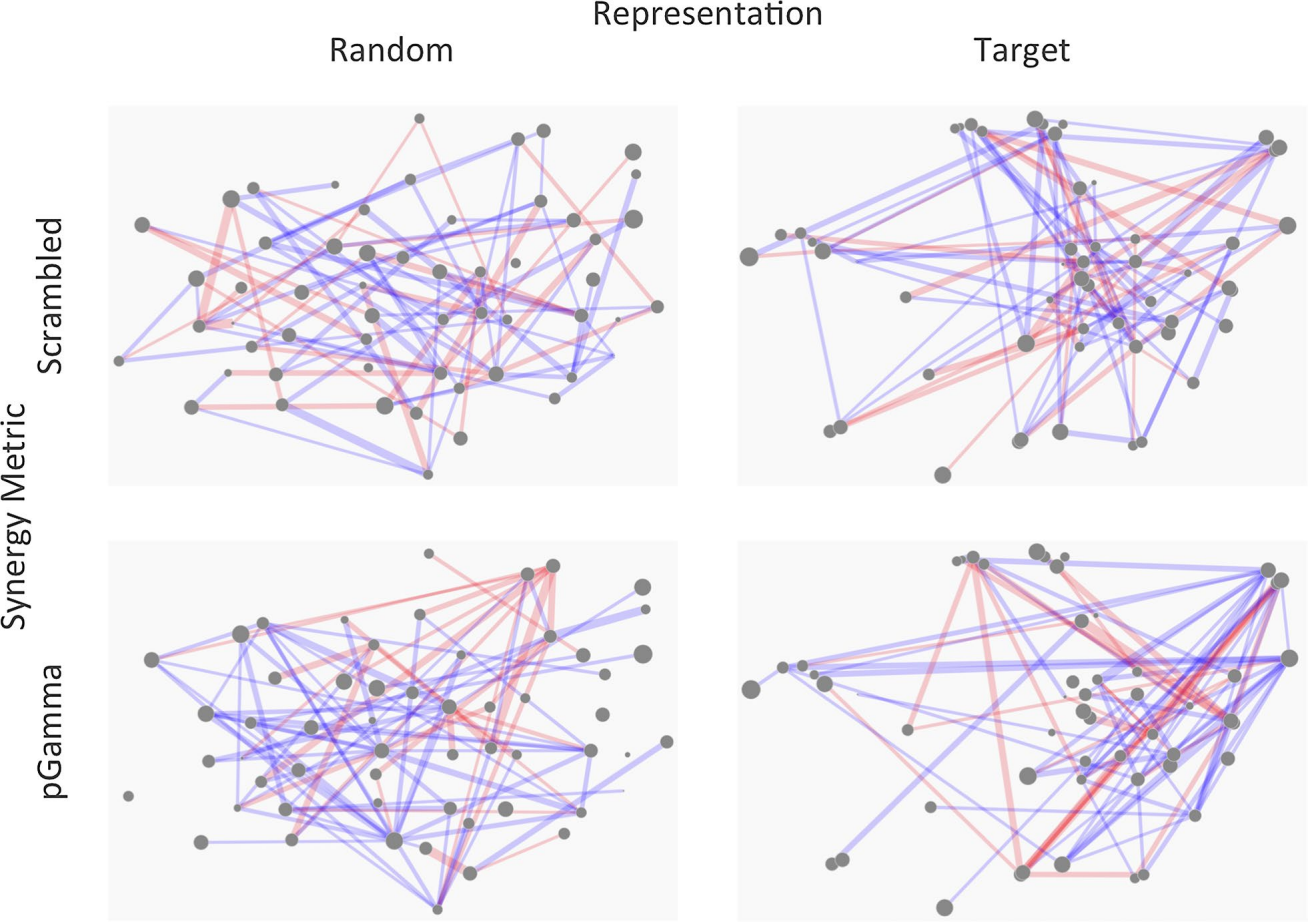
Validation through randomization and scrambling. In order to assess the meaningfulness of potential hypotheses drawn from a synergy map, it is advantageous to compare with random data, such as those in the figure, to protect against spurious correlations being interpreted for more than they are. The random compound positions (*leftmost maps*) are generated using random feature vectors, which were then reduced using t-SNE in an identical fashion to the Bayes Affinity vectors (*rightmost maps*). The random positioning of compounds appears not to produce the clusters observed when real data is used. Randomly shuffling the combinations values (*topmost maps*) reveals more realistic maps—there are several clusters which appear to share many synergies, for which hypotheses may have been proposed, illustrating the danger of overinterpretation of synergy maps.

In the authors’ opinion, the observations described above are much less clear in the heatmap or network visualization of the data, illustrating the strength of synergy maps. However there are some problems that arise, principally in ‘over fitting’ an interpretation—trends may appear at random, and as such ‘control’ visualizations should be consulted, to provide a reality check. These can be done by scrambling compound or combination data, or using random feature representations to generate compound coordinates, as shown in Fig. 8. Observed trends should certainly be treated with healthy skepticism, although it is likely that with the growth of high quality datasets, these chance correlations will lessen and more may be gained from the approach.

## Conclusion

Synergy Maps, a novel method for visualization of a combination data set was presented, integrating combination-based information in a network, with compound-based information using a dimensionality reduced scatter-plot. An accompanying interactive visualization tool was also introduced, which enables fast and simple exploration and presentation of combination data. An all-pairs combination dataset assayed against *P. falciparum* was analyzed as an example, identifying several properties already reported in the literature.

## Availability and requirements

Project name: Synergy Maps.

Project home page: https://www.github.com/richlewis42/synergy-maps.

Operating system(s): Platform independent/Google Chrome.

Programming language: Javascript and Python.

License: MIT.

## Abbreviations

FBA: flux balance analysis
PCA: Principal Components Analysis
MDS: Multi Dimensional Scaling
t-SNE: t-distributed Stochastic Neighbour Embedding
JSON: JavaScript Object Notation
CSV: comma separated values
HDAC: histone deacetylase
PI3K: phosphoinoside 3-kinase
